# Scriptaid Improves Cashmere Goat Embryo Reprogramming by Affecting Donor Cell Pluripotency Molecule NANOG Expression

**DOI:** 10.3390/ani15071022

**Published:** 2025-04-02

**Authors:** Xiaoshu Zhe, Hairui Ma, Wenqi Zhang, Rui Ding, Fei Hao, Yuan Gao, Gumara Uri, Gellegen Jiri, Garangtu Jiri, Dongjun Liu

**Affiliations:** 1 State Key Laboratory of Reproductive Regulation and Breeding of Grassland Livestock, School of Life Sciences, Inner Mongolia University, Hohhot 010070, China; 22208021@mail.imu.edu.cn (X.Z.); hairui2352079507@126.com (H.M.); 32308107@mail.imu.edu.cn (W.Z.); 32408144@mail.imu.edu.cn (R.D.); feihao@imu.edu.cn (F.H.); 21908009@mail.imu.edu.cn (Y.G.); 2 Alxa League Animal Quarantine Technology Service Center, Inner Mongolia, Alxa 750300, China; 3 Etoqqi Agricultural and Animal Husbandry Technology Extension Center, Inner Mongolia, Ordos 016100, China; m18847352613@163.com (G.U.); 18747713273@163.com (G.J.); 15048725403@163.com (G.J.)

**Keywords:** SCNT, Scriptaid, NANOG, histone acetylation, cashmere goat

## Abstract

Currently, the efficiency of somatic cell nuclear transfer (SCNT) technology is relatively low, primarily owing to reprogramming abnormalities in donor cells or reconstructed embryos. Using histone deacetylase inhibitor (HDACi) to artificially alter the epigenetic modifications of donor cells and improve the reprogramming ability of reconstructed embryos is effective in improving nuclear transfer efficiency. In this study, we used Albas cashmere goat cells as donor cells, treated them with Scriptaid, and constructed embryos using SCNT. The results suggest that donor cell treatment with Scriptaid significantly increased the cellular histone acetylation modification level, perturbed the expression of the pluripotency molecule NANOG, altered the reprogramming ability of embryos, and increased the developmental rate of SCNT-reconstructed embryos. Scriptaid inhibited donor cell proliferation, induced apoptosis, and blocked the G0/G1 phase of the cell cycle. These results provide a new research direction for improving SCNT efficiency and a new perspective in the fields of regenerative medicine, agriculture, and animal husbandry.

## Simple Summary

Somatic cell nuclear transfer (SCNT) is a commonly used biological technique that has been successfully used to clone a variety of mammals. However, the efficiency of SCNT technology is relatively low at present, and the reprogramming abnormality of donor cells or reconstructed embryos is the main reason for its low efficiency. Currently, the use of small-molecule compounds to artificially alter the epigenetic modifications of donor cells and improve the reprogramming ability of reconstructed embryos has become an effective way to improve the efficiency of nuclear transfer. In this study, we treated donor cells with Scriptaid, a histone deacetylase inhibitor (HDACi), and found that it perturbed the expression of pluripotency genes in donor cells, improved the reprogramming ability of reconstructed embryos, and affected the development rate of cloned embryos. SCNT has been widely used in the fields of regenerative medicine, agriculture, and animal husbandry, and the results of this study provide new insights into understanding and applying cell reprogramming technology.

## 1. Introduction

In 1996, the world’s first cloned sheep, Dolly, was created, marking the beginning of the era of somatic cell cloning. Extensive research has enabled the successful cloning of many somatic cell types from goats, pigs, cows, and other mammals using various somatic cells as donor cell sources [1]. Somatic cell nuclear transfer (SCNT) involves the transfer of somatic cells to enucleated oocytes, which are cultured and developed into new embryos and, subsequently, into individuals to produce cloned animals with the same genotype as that of the donor cells. However, SCNT technology has the disadvantage of low inefficiency [2]. Although the efficiency of the SCNT technique has improved in studies involving large mammals, the overall success rate remains low, typically ranging from 0.1% to 16% [3], limiting its application. Double homeobox (Dux) overexpression rescues the aberrant histone H3 lysine 9 acetylation (H3K9ac) levels and ensures normal genome activation during the 2-cell period, improving SCNT efficiency [4].

In mammalian studies, it is common to use relevant agents to directly modulate gene control and other methods that alter epigenetic modifications to change the dysfunctional developmental process of cloned embryos [5] and improve their reprogramming ability in vivo and in vitro. Histone deacetylase inhibitors (HDACi) have become common research agents for rescuing molecular modification levels by decreasing histone deacetylase (HDAC) activity and increasing histone acetylation levels, promoting gene transcriptional expression [6]. Scriptaid, a synthetic low-toxicity HDACi, has advantages over traditional HDACi (such as trichostatin A), including low-dose use, low toxicity, and short treatment duration. It directly enhances histone acetylation levels, promotes the function of reprogramming-associated transcription factors [7], interferes with the methylation of imprinted genes [8], and has a common application in somatic cell cloning research.

In this study, we investigated the functionality of HDACi in the SCNT process by altering the epigenetic modification capacity of donor cells cultured with Scriptaid in vitro and evaluated the effects of Scriptaid treatment on cell proliferation and apoptosis, as well as on the cell cycle of donor cells. Similarly, we elucidated the key pluripotency genes involved in the embryonic developmental process after Scriptaid treatment of donor cells during embryonic development. The results provide an experimental basis for selecting suitable donor cells for future SCNT in cashmere goats and offer a direction for establishing nuclear transplantation technology and practicing mammalian animal husbandry.

## 2. Materials and Methods

### 2.1. Cell Lines and Cell Cultures

The cell lines used in this experiment were goat fetal fibroblasts (gFFCs), goat adipose-derived stem cells (gADSCs), and goat muscle-derived satellite cells (gMDSCs), which were derived from Albas chamois goats. Goat fetal fibroblasts (gFFCs) were cultured in DMEM/F12 culture medium containing 10% fetal bovine serum (FBS), gADSCs were cultured in DMEM/F12 culture medium containing 20% FBS, gMDSCs were cultured in DMEM/F12 culture medium containing 20% FBS and 10% equine serum, they and were incubated at a 37 °C and 5% CO_2_. All cells used in this experiment were fifth-generation (passage) cells. The culture medium was changed every two days, and when the wall-adherent cells reached 80–90% confluence, the cells were enzymatically detached using 0.25% trypsin for 3 min to facilitate passaging. Scriptaid was dissolved in DMSO, and an aliquot was frozen and stored at −20 °C. Experimental cells with 70% confluence were treated with single-dose administration of Scriptaid at concentrations of 250, 500, and 750 nM for 24, 48, and 72 h, respectively, and cells without added Scriptaid were used as blank controls. Cell culture reagents were purchased from Vivacell (Shanghai, China).

### 2.2. CCK-8 Toxicity Assay

In this experiment, the cell activity of Scriptaid-treated cells was detected using CCK-8 (Yeasen, Shanghai, China) at different concentrations and time gradients. The cells were inoculated into 96-well plates and cultured for 24 h. Once the cells were fully adherent and confluent to 60%, they were cultured by adding Scriptaid in different concentrations. CCK-8 reagent was added to Scriptaid at 24 and 48 h, respectively, and cultured for 2 h in an incubator. The OD value at 450 nm was detected. The formula for calculating cell growth viability was as follows: cell viability* (%) = [A (dosing) − A (blank)]/[A (0 dosing) − A (blank)] × 100.

### 2.3. RNA Extraction and qRT-PCR

Total RNA was extracted from cells using RNAiso (Takara, Dalian, China) according to the manufacturer’s protocol, and cDNA was synthesized using the gDNA Removal Kit (Takara, Dalian, China) to remove any contaminating genomic DNA. According to the manufacturer’s instructions, cDNA was amplified for reverse transcription using TB Green^®^ Premix Ex Taq™ II (Takara, Dalian, China), and gene expression was assessed using the LightCycler 96 Real-Time Fluorescent Quantitative PCR Device System (Roche, Shanghai, China). Briefly, extracted RNA was added to enzyme-free water and incubated at 42 °C for 2 min, followed by the addition of RT mix and incubation at 37 °C for 15 min and 85 °C for 5 s. The qPCR reaction conditions were as follows: an initial denaturation step at 95 °C for 30 s, followed by 40 cycles of two-step PCR at 95 °C for 5 s and 60 °C for 34 s. Relative gene expression levels were calculated using the comparative CT method (2^−∆∆CT^) with gluceraldhédyde-3-phosphatase dehydrogenase (*GAPDH*) as the housekeeping gene. Table S1 shows the primer sequences for qRT-PCR.

### 2.4. Protein Extraction and Western Blot

Cell samples were extracted using the Mammalian Whole Protein Extraction Kit (CWBIO, Beijing, China) and Histone Extraction Kit (Epigentek, Farmingdale, NY, USA), while protein concentration was determined using a BCA Protein Assay Kit (Thermo Fisher Scientific, Waltham, WA, USA). Protein expression was detected by immunoblotting using Histone3 and α-tubulin as internal references. Proteins (20–30 µg) were separated by SDS-PAGE (90 V/30 min, 120 V/90 min) and electrophoretically transferred to nitrocellulose membranes, which were saturated with 5% skimmed milk for 1 h at room temperature and then incubated with primary antibody at 4 °C overnight. After three washes with TBST (CWBIO, Beijing, China), the membranes were incubated with HRP-coupled secondary antibody for 1 h at room temperature. Antigen–antibody complexes were visualized using a Pierce ECL protein blotting substrate (Thermo Fisher Scientific), while membrane images were acquired using a Tanon 4800 microscope, and analyzed in grayscale using ImageJ software (version 1.8.0). Table S2 shows the antibody resources.

### 2.5. EdU Cell Proliferation Assay

Cells were inoculated in 4-well plates at 1 × 10^4^ cells per well, with cells in the untreated medium used as the control group and cells in the drug-supplemented medium used as the experimental group. Cells were treated according to the instructions provided with the EdU Cell Proliferation Assay Kit (Ribobio, Guangzhou, China). Briefly, 100 µL of 50 µM EdU medium was added to each well, and the plates were incubated for 2 h, after which the medium was discarded and 50 µL of 4% paraformaldehyde (Solarbio, Beijing, China) was added and fixed overnight at room temperature. The next day, paraformaldehyde was discarded, 50 µL of 2 mg/mL glycine was added, and the mixture was shaken for 5 min. Glycine was discarded, 100 µL of PBS was added, and the mixture was shaken for 5 min. The plates were incubated with 0.5% TritonX-100 in a PBS shaker and permeabilized for 10 min, then the liquid was discarded, and the plates were washed with PBS for 5 min. Each well was incubated with 100 µL of 1× Apollo staining solution, and the plates were incubated at room temperature in the dark for 30 min. Then, 100 µL of 0.5% TritonX-100 was added and permeated for 30 min at room temperature. The permeabilized solution was discarded, 100 µL of PBS was added, and the plates were washed with PBS for 5 min. Next, 100 µL of 1× Hoechst33342 reaction solution was added to the plates, which were then incubated for 30 min, and the results were observed using a confocal microscope.

### 2.6. Flow Cytometry

Briefly, cells were inoculated in 6-well plates at a concentration of 5 × 10^4^ cells/well, with normal untreated cultures as a control. The cell cycle was detected using the Cell Cycle and Apoptosis Detection Kit (7sea Biotech, Shanghai, China), and the cells were fixed using 70% ethanol at 4 °C overnight. The fixed samples were centrifuged at 1000× *g* for 5 min, resuspended in 1 mL of pre-cooled PBS, and centrifuged to obtain the precipitate. We added 200 µL of PI stain, and the cells were gently resuspended and incubated at 37 °C for 30 min, and fluorescence was detected at 488 nm. Apoptosis was detected using an Annexin V-FITC/PI double staining kit (7sea Biotech, Shanghai, China). After the specified time, the cells were collected in 1.5 mL centrifuge tubes, resuspended in 400 µL of 1× binding buffer, and 5 µL of Annexin-V FITC staining solution was added, blown up, and mixed well. The cells were then incubated for 15 min at room temperature and protected from the light. Then, 10 µL of PI staining solution was added, blown up, and mixed well, and the cells were incubated in an ice bath and protected from light for 5 min. The stained samples were subjected to flow cytometry to detect FITC, PI, and FITC within 30 min. The samples were stained for FITC and PI fluorescence by flow cytometry within 30 min. The data were further analyzed using FlowJo software V10.

### 2.7. Somatic Cell Nuclear Transfer

The ovaries of velvet goats were mechanically dissected, and cumulus–oocyte complexes (COCs) were collected and cultured in M-199 maturation medium (Gibco, Grand Island, NY, USA) at 38.5 °C in a humidified atmosphere of 5% CO_2_ for 18 h. Following maturation, COCs were treated with 0.1% hyaluronidase solution (Sigma-Aldrich, St. Louis, MO, USA) under a stereomicroscope to remove the surrounding cumulus cells. The polar body and a small portion of the adjacent cytoplasm were aspirated using micromanipulation techniques. Donor cells treated with Scriptaid were suspended in a cytochalasin B droplet and microinjected into the perivitelline space of enucleated oocytes. The reconstructed embryos were incubated at 38.5 °C in 5% CO_2_ for 20 min. Electrofusion was performed using an ECM 2001 Electrocell Manipulator (Harvard Apparatus, Boston, MA, USA). Successfully fused embryos were activated in synthetic oviduct fluid supplemented with amino acids (SOFaa) containing 5 µM calcium ionophore A23187 for 5 min, followed by further activation in SOFaa with 2 mM 6-dimethylaminopurine for 3.5 h. Finally, the reconstructed embryos were transferred into an embryonic development medium and cultured at 38.5 °C in 5% CO_2_ for 48 h. Embryo development rates were calculated based on the proportion of embryos that reached the desired developmental stage.

### 2.8. Statistical Analysis

Data were analyzed using SPSS 26.0 software (IBM) and are presented as mean ± standard deviation (SD); all data were normally distributed. The differences were compared using Student’s *t*-test, and the *p*-value was determined using SPSS 26.0 software. *p* < 0.05 was considered statistically significant,* *p* < 0.05, ** *p* < 0.01, *** *p* < 0.001, **** *p* < 0.0001. All experiments were independently repeated thrice. GraphPad Prism 10 was used to construct the statistical and reproducibility plots.

## 3. Results

### 3.1. Scriptaid Suppresses Cell Proliferation in Dose- and Time-Dependent Fashion

**Figure 1:**
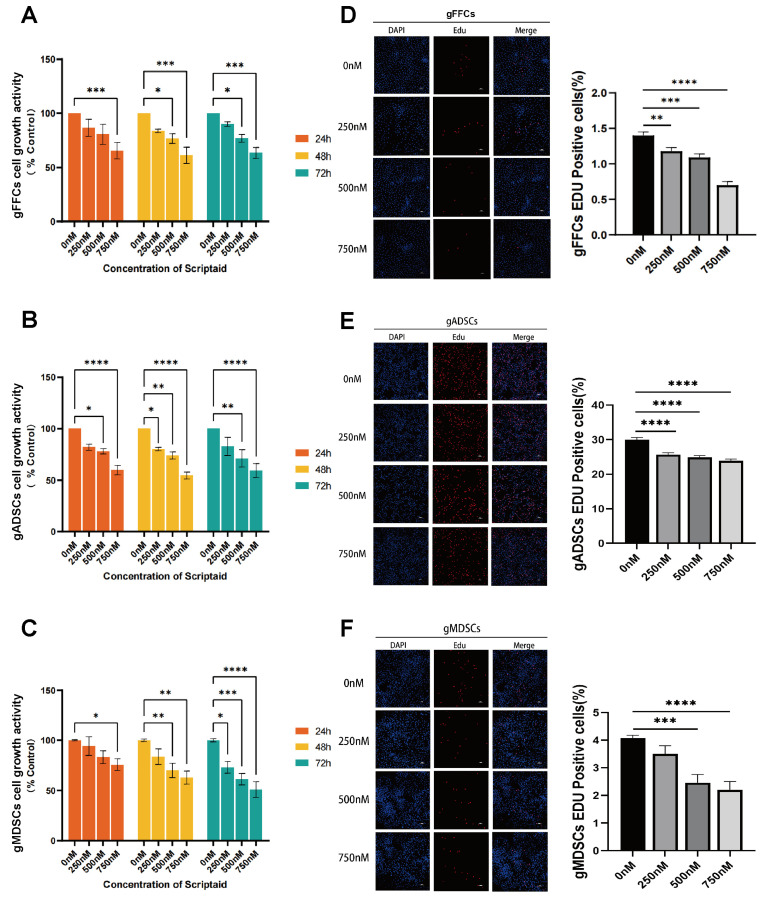
The effect of Scriptaid on cell growth activity and proliferation. (**A**) CCK8 assay for the effect of Scriptaid on the growth activity of gFFCs, with drug concentration in the horizontal coordinate and cell viability in the vertical coordinate. (**B**) CCK8 assay for the effect of Scriptaid on the growth activity of gADSCs. (**C**) CCK8 assay for the effect of Scriptaid on the growth activity of gMDSCs. (**D**) Statistical analysis of proliferation and cell proliferation rate of gFFCs by EdU assay. (**E**) Statistical analysis of proliferation and cell proliferation rate of gADSCs by EdU assay. (**F**) Statistical analysis of cell proliferation and cell proliferation rate of gMDSCs detected by EdU. Data (*n* ≥ 3) are represented as the mean ± SD. * *p* < 0.1, ** *p* < 0.01, *** *p* < 0.001 and **** *p* < 0.0001.

In the process of SCNT, the chromatin of the donor cell undergoes reprogramming to obtain totipotency; however, the proliferative capacity of donor cells affects the chromatin structure and reprogramming efficiency. To determine the toxicity of Scriptaid in cells, we selected three types of SCNT donor cells (gFFCs, gADSCs, and gMDSCs) that are commonly used in laboratories. Based on the experimental data of No JG et al. (2018) [9], we treated the cells with different concentrations of Scriptaid (0, 250, 500, and 750 nM) for different time periods (24, 48, and 72 h). We examined the effect of Scriptaid treatment on cell activity using the CCK8 assay, with the untreated group (0 nM) serving as the control. The results (Figure 1A–C) showed that the higher the Scriptaid concentration, the lower the cell viability, and the longer the drug treatment time, the more significant the difference. In addition, we examined the effects of treatment with different Scriptaid concentrations for 48 h on cell proliferation using the EdU assay. The results (Figure 1D–F) showed that the higher the Scriptaid concentration, the slower the cell proliferation, and that Scriptaid reduced the proliferation ability of the cells in a dose-dependent manner. These data indicate that Scriptaid significantly inhibited cell growth in a concentration- and time-dependent manner. Although the drug notably inhibited cell proliferation, there was no significant mutation or death during the experiment, possibly because of the high viability and low generation of donor cells or the relatively low toxicity of Scriptaid.

### 3.2. Scriptaid Regulates Alterations in Epigenetic Modifications

**Figure 2:**
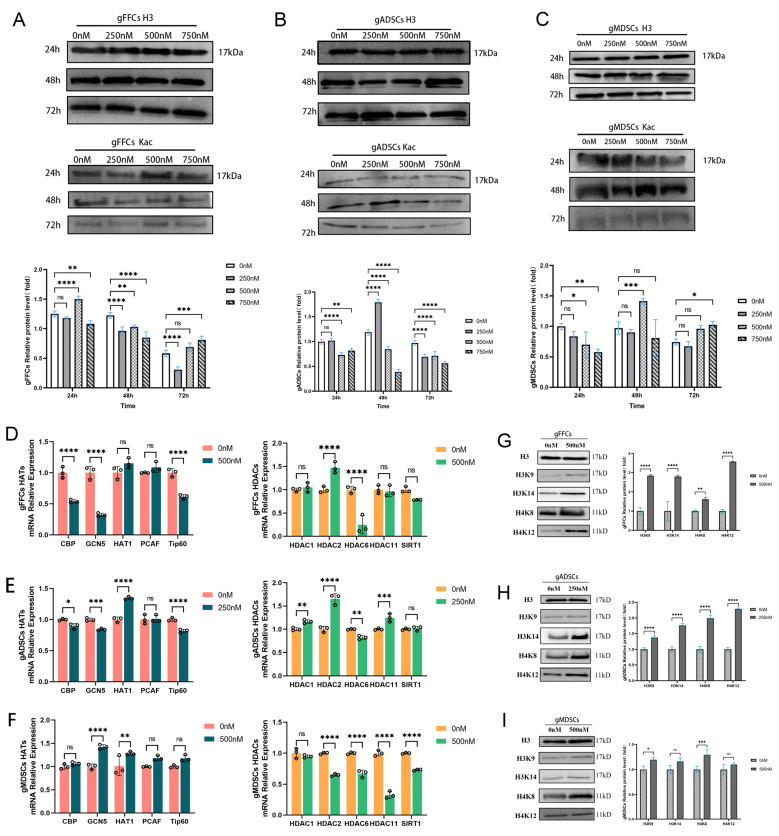
The effect of Scriptaid on histone acetylation levels in nuclear donor cells. (**A**) WB of Kac protein expression in gFFCs and grayscale analysis. (**B**) WB of Kac protein expression in gADSCs and grayscale analysis. (**C**) WB of Kac protein expression in gMDSCs and grayscale analysis. (**D**) mRNA level expression of HATs and HDACs in gFFCs. (**E**) mRNA level expression of HATs and HDACs in gADSCs. (**F**) mRNA level expression of HATs and HDACs in gMDSCs. (**G**) WB of histone acetylation site expression and grayscale analysis in gFFCs. (**H**) WB of protein acetylation site expression in gADSCs group and grayscale analysis. (**I**) WB of protein acetylation site expression in gMDSCs group and grayscale analysis. Data (*n* ≥ 3) are represented as the mean ± SD. ns > 0.1, * *p* < 0.1, ** *p* < 0.01, *** *p* < 0.001 and **** *p* < 0.0001.

In a developmental study of SCNT-reconstructed embryos, dynamic alterations in histone acetylation were found to be closely related to the zoom genetic algorithm (ZGA). High histone acetylation levels in donor cells enhance the developmental efficiency of SCNT embryos. In bovine [10], buffalo [11], and leopard cat [12] SCNT embryos, somatic histone hyperacetylation significantly increased the blastocyst rates. Scriptaid is a potent HDACi; however, its mechanism of action is unclear. To understand how Scriptaid affects histone acetylation levels, we first examined the effect of lysine-acetylated pan-antibody (Kac) on histone acetylation levels using Western blotting after treating cells with different concentrations (0, 250, 500, and 750 nM) of Scriptaid for different treatment time periods (24, 48, and 72 h). Each cell line was treated with unspiked (0 nM) protein as a control and H3 as the internal reference protein. The results showed that histone Kac levels were significantly altered after treatment with Scriptaid. The histone Kac levels were significantly higher in gFFCs when the cells were treated with 500 nM Scriptaid for 24 h (Figure 2A); however, with increasing treatment time and drug concentration, the acetylation levels decreased. Similarly, in gADSCs, the histone Kac levels were significantly higher than those in the other treated groups after treatment with 250 nM Scriptaid for 48 h (Figure 2B). In gMDSCs, following treatment with 500 nM Scriptaid for 48 h, the histone Kac levels were significantly higher than those in the other treated cells (Figure 2C). The same phenomenon was observed for gADSCs and gMDSCs: the higher the drug concentration and the longer the drug treatment time, the lower was the drug effect. High Scriptaid concentrations may be toxic to cells and affect their normal physiological functions, which may indirectly affect changes in acetylation levels. In our previous study, we demonstrated that the cellular response to Scriptaid significantly inhibited cell growth in a concentration- and time-dependent manner. Therefore, for subsequent analyses, we selected conditions where both had relatively little effect on the proliferative capacity of the donor cells and caused significant changes in histone acetylation levels. Combining the results of the study on the effect of Scriptaid on cell activity and the expression level of histone Kac, we finally chose the concentration and duration of Scriptaid treatment of gFFCs, gADSCs, and gMDSC as 500 nM for 24 h, 250 nM for 48 h, and 500 nM for 48 h, respectively.

Histone acetylation modification is jointly catalyzed by HDAC and HAT [13]; however, the mechanism by which Scriptaid regulates the alteration in histone acetylation levels and the enzymes that participate in this process are unknown. To elucidate these, we treated the cells with Scriptaid at the concentrations and times determined in the experiments described above. The differential expression of genes related to the histone acetylation transferase (HAT) (*CBP*, *GCN5*, *HAT1*, *PCAF*, and *Tip60*) and histone deacetylase (HDAC) (*HDAC1*, *HDAC2*, *HDAC6*, *HDAC11*, and *SIRT1*) families at the mRNA level in Scriptaid-treated cells was determined using qRT-PCR, with the untreated group as the control and *GAPDH* as the internal reference gene. The results showed that in gFFCs (Figure 2D), *PCAF* expression levels were significantly elevated, whereas those of *CBP*, *GCN5*, and *Tip60* were significantly reduced. *HDAC2* expression was significantly elevated, whereas that of *HDAC6* was significantly decreased. In gADSCs (Figure 2E), *HAT1* expression levels were elevated, whereas those of *CBP*, *GCN5*, and *Tip60* were significantly decreased. The expression levels of *HDAC2*, *HDAC1*, and *HDAC11* were significantly elevated, whereas those of *HDAC6* were decreased. In gMDSCs (Figure 2F), *GCN5* and *HAT1* showed increased expression, whereas *HDAC2*, *HDAC6*, *HDAC11*, and *SIRT1* exhibited significantly decreased expression. Notably, Scriptaid significantly increased the expression of the adult stem cell histone acetylase *HAT1* transcript, whereas the expression of the histone deacetylase HDAC6 in multiple cell types significantly decreased. In addition, Scriptaid had a more pronounced effect on histone acetylation levels in gMDACs, with elevated expression levels of almost all HAT family-related genes and decreased expression levels of all HDAC family-related genes.

To elucidate the effect of Scriptaid on histone acetylation, we analyzed the differences in the acetylation levels of histone sites (H3K9, H3K14, H4K8, and H4K12) in Scriptaid-treated cells using Western blotting, with the untreated group serving as the control and H3 as the internal reference. The results showed that the acetylation levels of the histone sites H3K9, H3K14, H4K8, and H4K12 were significantly increased in gFFCs (Figure 2G) and gADSCs (Figure 2H), and that of histone site H4K8 was significantly increased in gMDSCs (Figure 2I). These results indicate that Scriptaid significantly inhibits H4K8 deacetylase.

### 3.3. Scriptaid Perturbation of Pluripotency Molecule NANOG Expression

**Figure 3:**
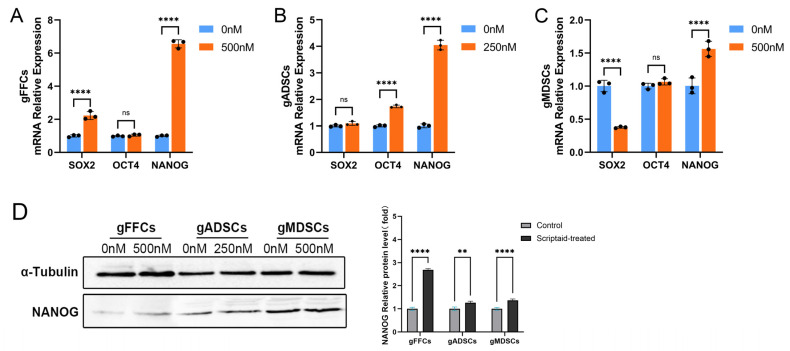
Effects of Scriptaid on pluripotency genes in nuclear donor cells. (**A**) qRT-PCR detection of mRNA expression level of pluripotency-related genes in gFFCs. (**B**) qRT-PCR detection of mRNA expression level of pluripotency-related genes in gADSCs. (**C**) qRT-PCR detection of mRNA expression level of pluripotency-related genes in gMDSCs. (**D**) WB detection of NANOG protein expression level and grayscale analysis. Data (n ≥ 3) are represented as the mean ± SD. ns > 0.1, ** *p* < 0.01 and **** *p* < 0.0001.

During SCNT, the donor cell nucleus requires complete reprogramming, and pluripotency genes, as the core of cellular reprogramming, play an important role in this process. The introduction of pluripotency genes into cells has been proposed to improve the reprogramming efficiency and developmental potential of embryos [14]. To determine the alterations in the expression of genes that are markers of cell reprogramming after Scriptaid treatment of donor cells cultured in vitro, we examined the expression levels of *OCT4*, *SOX2*, and *NANOG* mRNAs using qRT-PCR. Using the untreated group as a control, after Scriptaid treatment, the expression levels of *SOX2* and *NANOG* were significantly elevated in gFFCs (Figure 3A), whereas those of *OCT4* showed no significant differences. In gADSCs (Figure 3B), the expression levels of *OCT4* and *NANOG* were significantly elevated; however, SOX2 expression did not differ significantly. In gMDSCs (Figure 3C), *SOX2* expression was significantly decreased, *NANOG* expression was significantly elevated, and *OCT4* expression was not significantly affected. Subsequently, using Western blotting, we analyzed the expression levels of *NANOG* protein after Scriptaid treatment of the donor cells cultured in vitro and found that Scriptaid significantly increased the expression of *NANOG*, a pluripotency molecule (Figure 3D).

### 3.4. Scriptaid Induces Apoptosis and Blocks Cell Cycle Progression

During SCNT, *NANOG* expression is critical for the self-renewal and maintenance of pluripotency of embryonic stem cells, which, together with *OCT4* and *SOX2*, constitutes the core regulatory network of embryonic stem cell pluripotency and affects cell proliferation and differentiation [15]. *NANOG* promotes cell cycle progression by regulating the expression of cell cycle-related genes, inhibiting apoptosis, and protecting cells from programmed death, thereby supporting cell survival and proliferation. *NANOG* silencing or knockdown causes cell cycle arrest and inhibits stem cell growth [16]. Therefore, we investigated the effects of Scriptaid treatment on apoptosis and cell cycle alterations.

**Figure 4:**
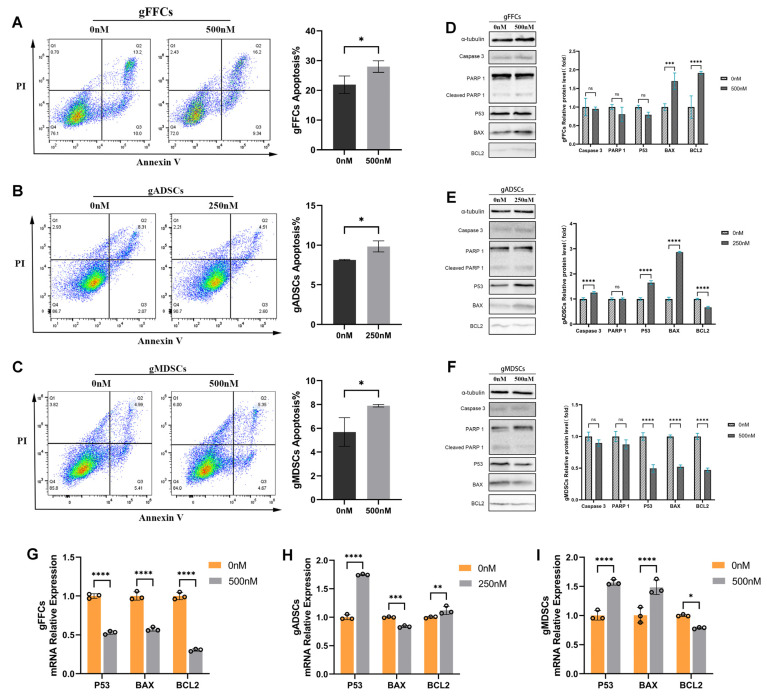
Effect of Scriptaid on apoptosis in donor cells. (**A**–**C**) Flow cytometry of Annexin V-FITC/PI staining to detect the apoptosis rate of gFFCs, gADSCs, and gMDSCs. (**D**–**F**) Western blot detection of apoptosis-related protein expression of gFFCs, gADSCs, and gMDSCs and grayscale analysis. (**G**–**I**) qRT-PCR to detect the expression of mRNA level of apoptosis-related genes in gFFCs, gADSCs and gMDSCs. Data (n ≥ 3) are represented as the mean ± SD. ns > 0.1, * *p* < 0.1, ** *p* < 0.01, *** *p* < 0.001 and **** *p* < 0.0001.

We performed flow cytometry based on Annexin V-FITC/PI staining in the untreated and Scriptaid-treated groups. The results (Figure 4A–C) showed that the percentage of apoptotic cells was significantly increased after Scriptaid treatment. To elucidate the mechanism of Scriptaid-induced apoptosis, we used Western blotting to determine the expression of apoptosis-related proteins, including cysteine aspartate aminotransferase family *Caspase-3*, *PARP1*, oncogene *P53*, pro-apoptotic protein *BAX*, and anti-apoptotic protein *BCL2*. In gFFCs (Figure 4D), the expression levels of *Caspase-3*, *PARP1*, and *P53* were decreased in Scriptaid-treated cells compared with those in the control group; however, the difference was not significant, and *BAX* and *BCL2* expression levels were significantly elevated. In gADSCs (Figure 4E), *Caspase-3* was activated with an upregulated expression, *PARP1* expression was not significantly changed, *P53* expression was significantly elevated, *BAX* expression was significantly elevated, and *BCL2* showed downregulated expression. In gMDSCs (Figure 4F), *PARP1* expression was not significantly changed; however, the expression levels of *P53*, *BAX*, and *BCL2* were significantly decreased. Furthermore, qRT-PCR showed that in gFFCs (Figure 4G), *P53*, *BAX*, and *BCL2* showed significantly decreased expression levels following Scriptaid treatment compared with those in the control. In gADSCs (Figure 4H), the expression levels of *P53* and *BCL2* were significantly elevated, whereas *BAX* expression was significantly decreased. In gMDSCs (Figure 4I), *P53* and *BAX* expression levels were significantly elevated, whereas *BCL2* expression was decreased. Although all apoptosis-related genes showed significantly altered expression patterns in Scriptaid-treated cells, the mechanisms of action differed among the cell lines, suggesting that Scriptaid may induce apoptosis in donor cells through multiple pathways.

**Figure 5:**
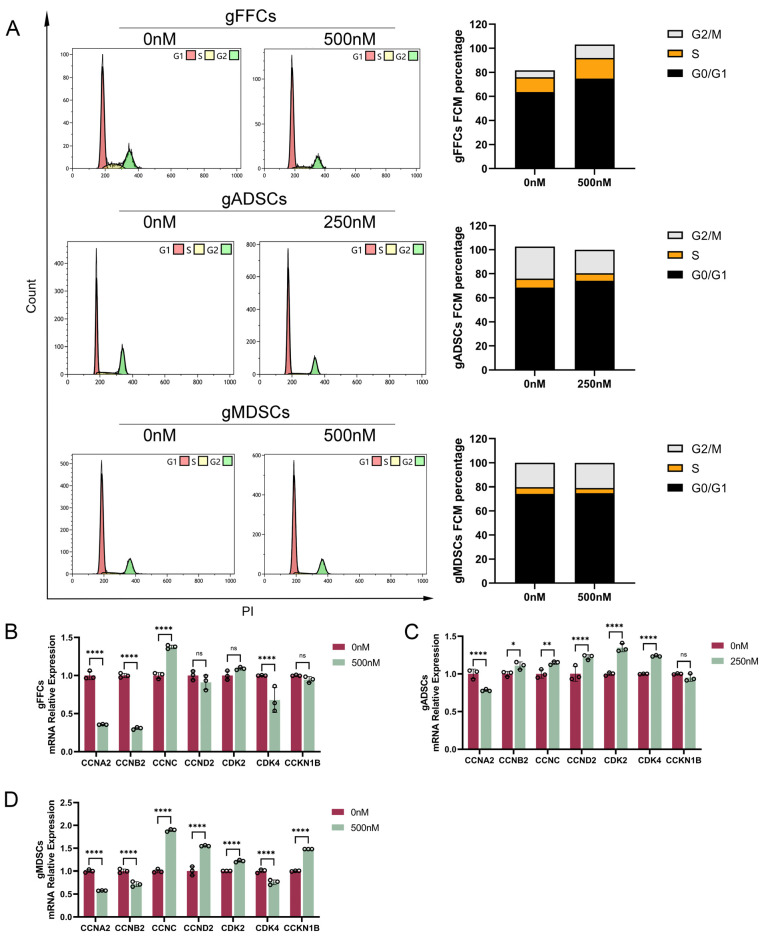
Effects of Scriptaid on the donor cell cycle. (**A**) Flow cytometry to detect the cell cycle of gFFCs, gADSCs, gMDSCs and statistical analysis of each period. (**B**–**D**) qRT-PCR to detect the expression of cycle-related gene mRNA levels in gFFCs, gADSCs, and gMDSCs. Data (n ≥ 3) are represented as the mean ± SD. ns > 0.1, * *p* < 0.1, ** *p* < 0.01 and **** *p* < 0.0001.

Furthermore, we performed cell cycle-related validations. Cell cycle analysis was performed using flow cytometry. The results (Figure 5A) showed that compared with the control group without drug treatment, the proportion of cells in the G0/G1 phase in gFFCs and gADSCs was elevated in the Scriptaid-treated cells; the proportion of cells in the G2/M phase was elevated in gFFCs, whereas it was decreased in gADSCs. Scriptaid-treated gMDSCs did not show any significant changes in the cell cycle. To further investigate the effect of Scriptaid on the cell cycle, we evaluated the differences in the mRNA expression levels of cycle-related genes (G0/G1 phase: *CDK2*, *CDK4*, *CCNC*, and *CCND2*; S phase: *CCNA2*; G2/M phase: *CCNB2* and *CDKN1B*) using qRT-PCR. In gFFCs (Figure 5B), the expression levels of *CCNA2*, *CCNB2*, *CDK4*, and *CDKN1B* were significantly decreased, and those of *CCNC* were significantly increased after drug treatment compared to the control group. In gADSCs (Figure 5C), the expression levels of *CCNA2* and *CDKN1B* were significantly decreased after drug treatment, whereas those of *CCNB2*, *CDK4*, *CCNC*, *CDK2*, and *CCND2* were significantly elevated. In gMDSCs (Figure 5D), *CCNA2*, *CDKN1B*, and *CDK4* showed significantly decreased expression, whereas *CCNB2*, *CCNC*, *CDK2*, and *CCND2* exhibited significantly elevated expression after drug treatment. These results indicate that Scriptaid treatment of cells upregulates the expression of the G0/G1 phase-related genes *CDK2* and *CCNC* and downregulates the S phase-related gene *CCNA2*, blocking the cell cycle in the G0/G1 phase and affecting the entry of cells into the S and G2/M phases.

### 3.5. Scriptaid Treatment of Donor Cells Increases the Rate of SCNT Embryo Oviposition

It has been found that the Scriptaid treatment of porcine donor cells effectively improves the reprogramming ability of SCNT embryos. The developmental stages of SCNT-reconstructed embryos are usually classified according to the time of embryonic development. Within 12–24 h after nuclear transfer in large mammals, the reconstructed embryos enter the oogamy stage and may form embryos at different stages, such as 2-cell, 4-cell, 8-cell, and other stages. Embryonic cells undergo complex cell cycle regulation, facilitating organ formation and embryonic development. Therefore, we selected the 24-hour time point to evaluate whether Scriptaid treatment of donor cells for nuclear transfer would affect the cleavage rate during embryonic development.

**Figure 6:**
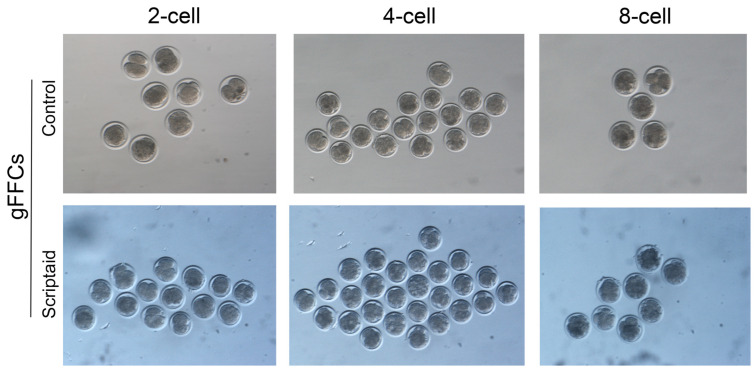
The SCNT reconstructed embryo development map.

We prepared gFFCs as SCNT donor cells for the experiment, which were divided into two groups: non-treated and treated (using 500 nM Scriptaid). The embryos were cultured in vitro through cloning operation, and after 24 h of culture, the oogenesis rate was evaluated. The results are shown in Table 1. The total embryo cleavage rate was 48.14% in the untreated cells and 64.58% in the Scriptaid-treated cells (*p* < 0.05), indicating that Scriptaid treatment of donor cells significantly increased the SCNT-reconstructed embryo cleavage rate. Although the developmental rate of the embryos in the 2-cell period was lower than that of the control group, the developmental rate of the reconstructed embryos after Scriptaid treatment was significantly higher than that of the control group in the 4–8-cell period. The results indicated that Scriptaid treatment of cells increased the ability of reconstructed embryos to develop toward the 4–8-cell period and significantly promoted the reprogramming ability of embryos. Embryos reconstructed after the Scriptaid treatment of oocytes and donor cells usually die or show uneven embryonic cleavage or zona pellucida ruptures. However, in our study, there was no significant effect on embryo quality after treating donor cells only with Scriptaid, and the reconstructed embryos developed well with uniformly spherical oval cleavage spheres (Figure 6).

## 4. Discussion

Mammalian SCNT has become a prominent technology in the field of reproductive and regenerative medicine as it has produced many high-quality and specific progeny in the last 20 years and is common in studies such as livestock breeding and germplasm resource conservation. Although the technology has been improved and explored, the low efficiency of SCNT cloning has limited its applications [17]. Abnormalities in the epigenetic reprogramming of donor cell nuclei or reconstructed embryos cause aberrant gene expression patterns [18,19], leading to low cloning efficiency, abnormal cloned embryo phenotypes, and low viability of cloned individuals. It has been demonstrated in mammalian SCNT-related studies that enhanced histone acetylation levels can promote gene transcript expression, which can increase the reprogramming capacity of donor cells and affect the development of SCNT embryos [20]. Reconstructed embryos show lower histone acetylation levels than those produced by in vitro fertilization (IVF) [15]. Therefore, the use of HDACi to treat donor cells or embryos to increase the acetylation level and regulate embryo development has become a commonly used tool.

Scriptaid has lower cytotoxicity and promotes higher acetylation levels than other HDACi. It is an HDAC6 selective inhibitor [21] that effectively inhibits histone deacetylation. Our results showed that Scriptaid treatment upregulated HAT1 expression and downregulated HDAC6 expression, consistent with the findings of previous studies. Scriptaid is more specific than general HDACi, such as TSA, suberoylanilide hydroxamic acid (SAHA), and valproic acid (VPA). Scriptaid has been reported to increase H4K12 acetylation levels [22]. However, our study showed that Scriptaid also increased H3K9, H3K14, and H4K8 acetylation levels, and the change in the H4K8 acetylation level was more targeted, which has not been reported in previous studies.

Pluripotency molecules are expressed in cells, enable cells to develop into multiple types, and are usually active during the early stages of mammalian embryonic development. Among these genes, increased expression levels of *SOX2*, *OCT4*, and *NANOG* are used as markers for the increased reprogramming capacity of embryos in vitro [23]. During SCNT, donor cells undergo specific epigenetic and gene expression changes to reprogram from one cell type to another to restore their pluripotency, and the expression levels of pluripotency molecules directly affect the developmental potential of SCNT embryos. The introduction of pluripotency molecules into SCNT donor cells has been proposed to improve the reprogramming efficiency and developmental potential of cloned embryos after nuclear transfer [24]. However, the expression of pluripotency molecules in donor cells is usually subject to epigenetic regulation, such as DNA methylation and histone modifications, affecting molecular expression [25], a critical step in embryo reprogramming. In this study, pluripotency molecular expression was elevated in adult stem cells after Scriptaid treatment, and only *NANOG* expression was significantly elevated in gFFCs, possibly because gFFCs are terminally differentiated cells and have low expression of *SOX2* and the endogenous factor *OCT4*. Also, significant changes in *OCT4* and *SOX2* gene expression occurred only in the pre-developmental stage of the reconstructed embryos. Scriptaid treatment increased the *NANOG* expression. This suggests that HDACi increases the acetylation level of *NANOG*, promotes the binding of transcription factors, and alters the reprogramming ability of the donor cells. Various epigenetic modifications regulate NANOG expression and function. Deoxyribonucleic acid methylation regulates the expression levels of *NANOG* by affecting intercellular contact downregulation [26], and histone acetylation affects *NANOG* expression by altering the chromatin structure [27].

*NANOG* inhibits apoptosis and protects stem cells from programmed cell death by inhibiting the P53 pathway and regulating the expression of *BCL2* family proteins [28]. The absence of NANOG significantly increases early and late apoptosis, which is mediated by *Caspase 3* activation [29]. In our study, the Scriptaid treatment of donor cells promoted apoptosis, and the mechanisms of action differed among the cells. This could be due to the altered expression of *NANOG*, which downregulates *BCL2* expression and regulates the expression of *BCL2* family proteins, leading to the upregulation of *P53* expression. This could also be because *P53* overexpression inhibits *BCL2* expression and activates *BAX*, leading to apoptosis. It is also possible that, similar to the report of Yao (2018) [30], the induction of apoptosis in Scriptaid-treated cells may result from the activation of elevated *P21* expression through a P53-independent mechanism. Silencing or knockdown of *NANOG* causes cell cycle arrest and inhibits stem cell growth. In vitro studies have shown that if donor cells are subjected to in vitro-induced cell cycle arrest in the G0/G1 phase, the reprogramming ability of donor cells is affected, and the developmental efficiency of nuclear-transplanted embryos is significantly improved [31]. Scriptaid treatment reduced the proportion of cells in the S phase and increased the proportion of cells in the G0/G1 or G2/M phase, inhibiting cell proliferation and the expression of relevant signaling pathways, and even inducing apoptosis [32], similar to the results of this study. Scriptaid treatment of donor cells caused the blockage of the G0/G1 phase cells and elevated CDK2 and CCNC expression, which affected cell entry into the S and G2/M phases, altering the reprogramming ability of the cells.

In a Scriptaid versus interspecific somatic cell nuclear transfer (iSCNT) study, no differences were found in terms of the 2-cell and developmental 4–8-cell stages after Scriptaid treatment of donor cells; however, the percentage of development up to the 16-cell stage was significantly higher. Scriptaid treatment increases blastocyst rates [33] and promotes full-term development of SCNT pig embryos [34]. In our study, Scriptaid treatment of donor cells had no significant effects at the 2-cell stage and caused a significantly increased cleavage rate when the embryos developed to the 4–8-cell stage. These phenomena may be due to the activation of the expression of the pluripotency gene *NANOG* in donor cells, which enhances the reprogramming potential of cloned embryos by affecting the level of epigenetic modifications of the cells, thus increasing the rate of reconstructed embryo development. However, in early embryonic studies on pigs, the expression pattern of *NANOG* in embryonic stem cells differed from that in mice, and species-specific regulation of *NANOG* expression may occur during SCNT reprogramming [35].

Therefore, Scriptaid has a significant functional mechanism that alters the epigenetic state and gene expression pattern of the pluripotency molecule *NANOG*, which helps cells acquire or restore pluripotency, affects cell proliferation, apoptosis, and cycle changes, and alters cell reprogramming ability to effectively increase the rate of reconstructed embryo oogenesis. This process reveals the significance of pluripotency genes in cell reprogramming and embryonic development and provides a new direction for mammalian cloning and regenerative medicine research.

## 5. Conclusions

Our study confirmed that in donor cells, the histone deacetylase inhibitor Scriptaid inhibits *HDAC6* expression, increases H4K8 acetylation levels, upregulates the expression of the pluripotency molecule *NANOG*, induces apoptosis, and blocks the cell cycle at the G0/G1 phase to increase the rate of SCNT-reconstructed embryo development. Scriptaid alters the reprogramming capacity of reconstructed embryos by modulating the epigenetic modifications of the donor cells, perturbing the expression of pluripotency-related molecules, and altering the reprogramming ability of reconstructed embryos.

## Figures and Tables

**Table 1:** Statistics on the development rate of SCNT embryos from Scriptaid-treated donor cells.

Treatment	No. Constructed Embryo	Cleavage Rate (%)	2-Cell Rate (%)	4-Cell Rate (%)	8-Cell Rate (%)
Control	324	48.14	50 a	30.76 a	19.23 a
Scriptaid	350	64.58	37.93 b	35.12 b	24.14 b

Cleavage rate: Cleaved embryos/reconstructed embryos, 2-cell rate: 2-cell/cleaved embryos, 4-cell rate: 4-cell/cleaved embryos, 8-cell rate: 8-cell/cleaved embryos. Within the same column, different superscripts indicate significant differences, *p* < 0.05.

## Data Availability

All data are presented in the manuscript.

## Supplementary Materials

The following supporting information can be downloaded at: https://www.mdpi.com/article/10.3390/ani15071022/s1, Table S1: Primer sequences for qRT-PCR. Table S2: Antibodies resources.

## Abbreviations

The following abbreviations are used in this manuscript:

SCNT	Somatic cell nuclear transfer
HDACi	Histone deacetylase inhibitor
Dux	Double homeobox
gFFCs	Goat fetal fibroblast cells
gADSCs	Goat adipose-derived stem cells
gMDSCs	Goat muscle-derived satellite cells
ZGA	Zoom genetic algorithm
Kac	Lysine-acetylated pan-antibody
HAT	Histone acetylation transferase
HDAC	Histone deacetylas
IVF	In vitro fertilization
iSCNT	Interspecific somatic cell nuclear transfer
DRB	5,6-Dichlorobenzimidazole 1-β-D-ribofuranoside
TSA	Trichostatin A
SAHA	Suberoylanilide hydroxamic acid
VPA	Valproic acid
COCs	Cumulus–oocyte complexes
