# Supplementary material for: Scriptaid Improves Cashmere Goat Embryo Reprogramming by Affecting Donor Cell Pluripotency Molecule NANOG Expression
Source: Animals (Basel). 2025 Apr 2;15(7):1022. doi: 10.3390/ani15071022 (PMC11988105; doi:10.3390/ani15071022)
Supplement: Supplementary file 1 [file animals-15-01022-s001.zip › animals-3508872-supplementary.pdf]

animals

Table S1. Primer sequences

Gene Name	Sequence	Product Length (bp)
GAPDH	F 5' GGTCGGAGTGAACGGAT 3'	100
	R 5' TCTGCCTTGACTGTGCC 3'	
P53	F 5' TCAGGAGACATTTTCCGACT 3'	100
	R 5' GAGGCTCTGGCATTTGG 3'	
BAX	F 5' TCCGACGGCAACTTCAA 3'	96
	R 5' ACAGGGACAGCAGGCAC 3'	
Bcl2	F 5' CCTGTGGATGACCGAGTA 3'	92
	R 5' GACAGCCAGGAGAAATCA 3'	
SOX2	F 5' CATGCACCGCTACGACG 3'	88
	R 5' GCCCTGCTGAGAATAGGACA 3'	
OCT4	F 5' CCAGAAGGGCAAACGAT 3'	98
	R 5' GGAATGGGACCGAAGAGTA 3'	
NANOG	F 5' GCAACGGCAGAATACCC 3'	100
	R 5' CATTGATTGTTCCAAGGCT 3'	
CCNA2	F 5' TAAAGTATTTGCCATCAGTTATC 3'	94
	R 5' GTAGGTCTGGTGAAGGTCC 3'	
CCNB2	F 5' GCACCCACCAAAACAAC 3'	100
	R 5' AGAGCAAGGCATCAGAAA 3'	
CCNC	F 5' GCTTTCTGTGGATATGGAGA 3'	96
	R 5' GAGGAGGTTTTGGTTTCG 3'	
CCND2	F 5' AGCAGAAGTGCGAAGAGG 3'	98
	R 5' TGATGGAGTTGTCGGTGTA 3'	
CDK2	F 5' CCCGCTGTGCTCCTATC 3'	96
	R 5' TGGGGAAACTTGGCTTA 3'	
CDK4	F 5' GGTGTCGGTGCCTATGG 3'	94
	R 5' GGTCAGTTCGGGCTGTT 3'	
CDKN1B	F 5' GTGCCTTTACTTGGGTCTC 3'	100
	R 5' GCAGGTCGCTTCCTTATC 3'	
HDAC1	F 5'GCCAAAGGGGTCAAGGAAG 3′	96
	R 5' CTGAAGGGTTGTGGGATAAAGG 3′	
HDAC2	F 5' CTTTCAACTGGTGGCTCA 3'	100
	R 5' CTTTAGTAACTCAAGGATGGC 3'	
HDAC6	F 5′ CCAGCGCATCCACTGTATCA 3′	189
	R 5′ TCATAGTTGGCACCCTCACG 3′	
HDAC11	F 5' GAAAATGGGGCAAAGTG 3'	100
	R 5' GAAGTTGGGCAGGAAGA 3'	
SIRT1	F 5′ ATCCTCTAGTTCTTGCGGCAGT 3′	94
	R 5′ TTCGTCATTCTCACTTTCATCCTC 3′	
CBP	F 5′ CTTGGACTCCCCTACCTG 3′	166
	R 5′ TGATGGCTGCTGATCTGT 3′	
GCN5	F 5' GGCATCATTGAGTTCCAC 3'	94
	R 5' CCAGAGTCTTGTGCTTCG 3'	
HAT1	F 5' TTACAGCGGAAGATCCAT 3'	90
	R 5' TCATAAACCCGTCTAGCAT 3'	
PCAF	F 5′ CAGGGGTGTGATCGAGTTCC 3′	167

	R 5′ GGGTTTTGTGTTTCGGGTCG 3′	
Tip60	F 5′ CGAAGCTACTGGTCCCAGAC 3′<br>R 5′ CCTCCGACAGGGTGAGGATA 3′	193

Table S2. Antibodies resources

Antibodies	Source	ID
GAPDH polyclonal antibody	Proteintech	10494-1-AP
Histone 3 polyclonal antibody	Proteintech	17168-1-AP
Alpha Tubulin Polyclonal antibody	Proteintech	16007-1-AP
NANOG Polyclonal antibody	Proteintech	14295-1-AP
BCL2 Polyclonal antibody	Proteintech	12789-1-AP
PARP1 Polyclonal antibody	Proteintech	13371-1-AP
Caspase 3/p17/p19 Polyclonal antibody	Proteintech	19677-1-AP
P53 Polyclonal antibody	Proteintech	10442-1-AP
Bax Polyclonal Antibody	Proteintech	50599-2-Ig
Anti-acetyllysine mouse mAb	PTM BIO	PTM-102
Anti-Acetyl-Histone H4 (Lys12) Rabbit pAb	PTM BIO	PTM-121
Anti-Acetyl-Histone H4 (Lys8) Rabbit pAb	PTM BIO	PTM-120
Anti-Acetyl-Histone H4 (Lys14) Rabbit pAb	PTM BIO	PTM-113RM
Anti-Acetyl-Histone H3 (Lys9) Rabbit pAb	PTM BIO	PTM-112
HRP-conjugated Goat Anti-Rabbit IgG(H+L)	Proteintech	SA00001-2
HRP-conjugated Goat Anti-Mouse IgG(H+L)	Proteintech	SA00001-1
